# Expression and clinical significance of the NEK7-NLRP3 inflammasome signaling pathway in patients with systemic lupus erythematosus

**DOI:** 10.1186/s12950-018-0192-9

**Published:** 2018-09-03

**Authors:** Zhen-Zhen Ma, Hong-Sheng Sun, Ji-Cai Lv, Lei Guo, Qing-Rui Yang

**Affiliations:** Department of Rheumatology and Immunology, Shandong Provincial Hospital Affiliated to Shandong University, Jinan, 250021 Shandong China

**Keywords:** Cytokines, Lupus nephritis, NEK7-NLRP3 inflammasome, Peripheral blood mononuclear cells, Systemic lupus erythematosus

## Abstract

**Background:**

The aim of the study was to investigate the expression of the NEK7-NLRP3 inflammasome signaling pathway in the peripheral blood mononuclear cells (PBMCs) of patients with systemic lupus erythematosus (SLE), as well as its clinical significance.

**Methods:**

A total of 38 SLE patients and 33 healthy volunteers were recruited. Real time PCR and western blotting were performed to determine mRNA and protein levels of *NEK7*, NLRP3 inflammasome components (*NLRP3*, *ASC*, and *Caspase-1*), and downstream cytokines (*IL-1b* and *IL-18*) in PBMCs from the two groups. ELISA was used to detect serum levels of *IL-1b* and *IL-18*. The same methods were used to detect changes in the above indices in the 25 SLE patients after treatment. Correlations between clinical and laboratory parameters were also analyzed.

**Results:**

Compared to those in healthy controls, levels of *NEK7, NLPR3,* and *ASC* were lower in SLE patients; however, *Caspase-1*, *IL-1b*, and *IL-18* were expressed at higher levels. mRNA levels of *NEK7*, *NLRP3*, and *ASC* were inversely correlated with disease activity, whereas a positive correlation was observed with *IL-1b* and *IL-18*. After treatment, mRNA levels of *NEK7* and *NLRP3* increased, whereas *Caspase-1*, *IL-1b*, and *IL-18* decreased significantly. Compared to those in SLE patients without renal damage, patients with lupus nephritis (LN) exhibited lower mRNA levels of *NEK7*, *NLRP3*, and *ASC* but higher levels of *Caspase-1*, *IL-1b*, and *IL-18*.

**Conclusions:**

Results indicate that the expression of the NEK7-NLRP3 complex might play a protective role in the pathogenesis of SLE and is inversely correlated with disease activity. A positive effect of *NEK7* on *NLRP3* was observed, and the low expression of *NLRP3* in SLE patients might be related to the low expression of *NEK7*. Overexpression of *Caspase-1* in SLE patients mediates the maturation and release of *IL-1b* and *IL-18*, and contributes to the pathogenesis of SLE and LN.

## Background

Systemic lupus erythematosus (SLE) is a chronic autoimmune disease involving multiple systems, organs, and autoantibodies. The incidence rate of SLE in various regions and diverse races is different. The overall incidence rate is 40–80/10 million individuals and the male-to-female ratio is 1:10, with this disease being more prevalent in young women of child-bearing age. At present, interactions among many factors such as heredity, environment, infection, immunity, and hormone levels, are closely related to the pathogenesis of SLE. Immune dysfunction plays an important role in the formation and development of SLE, and especially, the adaptive immune system has been the focus of many studies. Abnormal activation and dysfunction of T cells and B cells results in the production of high levels of autoantibodies, inflammatory cytokines, and circulating immune complexes, as well as their invasion into tissues and organs, causing widespread damage [1]. As the first barrier, the innate immune system plays an important role in the removal of foreign pathogens and the activation of an effective adaptive immune response. The innate immune system distinguishes pathogen associated molecular patterns (PAMPs) via pattern recognition receptors (PRRs). To date, three types of PRRs have been identified, of which, some NOD-like receptors (NLRs) can be activated by specific endogenous or exogenous stimuli and form a large protein complex referred to as the inflammasome [2]. Four inflammasome, namely NLRP1, NLRP3, IPAF, and AIM2, have been discovered, among which, the study of NLRP3 has been the most extensive and universal. The NLRP3 inflammasome can be activated by a variety of pathogens such as bacteria, viruses, metabolic toxins, saturated fatty acids, amyloid peptides, adenosine triphosphate, and urate salts, and is related to the development of many diseases [3]. It consists of three parts, specifically, *NLRP3*, *ASC*, and *Caspase-1*; activation of *NLRP3* further activates the inflammatory caspase protein kinase (*Caspase-1*), resulting in production of the active forms of the inflammatory cytokines interleukin-1b (*IL-1b*) and interleukin-18 (*IL-18*), which plays an important role in inflammation and the immune response.

Studies have found that in mouse models of lupus, the NLRP3-ASC-Caspase-1 signaling pathway is activated, and with a P2X7 receptor blocker (selective potassium channel inhibitor), the activity of the whole pathway was inhibited; thus, specific inhibition of the P2X7 receptor might represent a new direction for SLE therapy [4]. However, our previous studies found that NLRP3 inflammasome components were expressed at low levels in peripheral blood mononuclear cells (PBMCs) from patients with SLE, which was inversely correlated with disease activities, suggesting that expression of the NLRP3 inflammasome might be a protective factor for SLE patients [5]. This might be related to the following factors. First, the adaptive immune response system is activated during the pathogenesis of SLE, and T and B lymphocytes are activated in large quantities. It has been reported that upon the activation of adaptive immunity, NLRP3 inflammasomes are directly suppressed by T cells [6]. In contrast, the low expression of the NLRP3 inflammasome in PBMCs from SLE patients might be related to the high expression of type I interferon (IFN-I), which is common in SLE patients and murine models of lupus. IFN-I has a significant inhibitory effect on the activation of the NLRP3 inflammasome [7] and can inhibit its activation through the signal transducer and activator of transcription 1 (STAT1) pathway [8].

Recent research has revealed that NIMA-related kinase 7 (*NEK7*), a serine and threonine kinase involved in mitosis, acts as a key mediator of the activation of NLRP3 inflammasome signaling [9]. In this study, we investigated the potential role of the NEK7- NLRP3 inflammasome in the pathogenesis of SLE and its association with disease activity. We detected expression of the NEK7-NLRP3 inflammasome pathway in Chinese Han SLE patients at the mRNA and protein levels and investigated its clinical significance to determine if it could represent a new target for SLE treatment.

## Results

### Clinical characteristics of SLE patients and healthy controls

Thirty-eight SLE patients were recruited and the mean age was 35.68 years (range, 17–66); for the 33 healthy controls, the mean age was 34.73 years (range, 18–68). The two groups were well matched in terms of age, gender, and race. The main demographics and clinical features of SLE patients and healthy controls are summarized in Table 1.

**Table 1:** Demographic, clinical and laboratory characteristics of SLE patients and healthy controls

Variables	SLE patients *n* = 38	Healthy controls *n* = 33
Sex(Male / female)	5/33	4/29
Age, years	35.68 ± 10.24^a^	34.73 ± 12.50^a^
Duration of disease, months	43.93(0–180) ^b^	
Anti-ds-DNA antibody, RU/mL	308.95(10–1579) ^b^	
Anti-AunA antibody, RU/mL	90.45(2–200) ^b^	
C3, g/L	0.78(0.24–1.42) ^b^	
C4, g/L	0.16(0.06–0.47) ^b^	
IgA, g/L	2.95(0.86–11.70) ^b^	
IgG, g/L	16.19(2.35–67.50) ^b^	
IgM, g/L	1.06(0.33–2.57) ^b^	
IgE, IU/mL	240.34(18.50–1960) ^b^	
ESR, mm/h	46.14(2–107) ^b^	
CRP, mg/L	19.10(0.24–96.24) ^b^	
SLEDAI	8.36(0–18) ^b^	
LN (%)	13(37.14)	
24 h proteinuria, g/L	0.90(0–7.23) ^b^	

Notes: ^a^ means $\overline{x}\pm s$; ^b^ means “mean value” and (minimum-maximum); *Anti-dsDNA antibody* anti-double stranded deoxyribonucleic acid antibody, *Anti-AnuA antibody* anti-nucleosome antibody, *ESR* erythrocyte sedimentation rate, *CRP* c-reactive protein, *C3* complement 3, *C4* complement 4, *Igs* immunoglobulins, *SLEDAI* systemic lupus erythematosus disease activity index, *LN* lupus nephritis

### mRNA levels of *NEK7*, NLRP3 inflammasome components, and downstream cytokines in PBMC from SLE patients and healthy controls

**Fig. 1:**
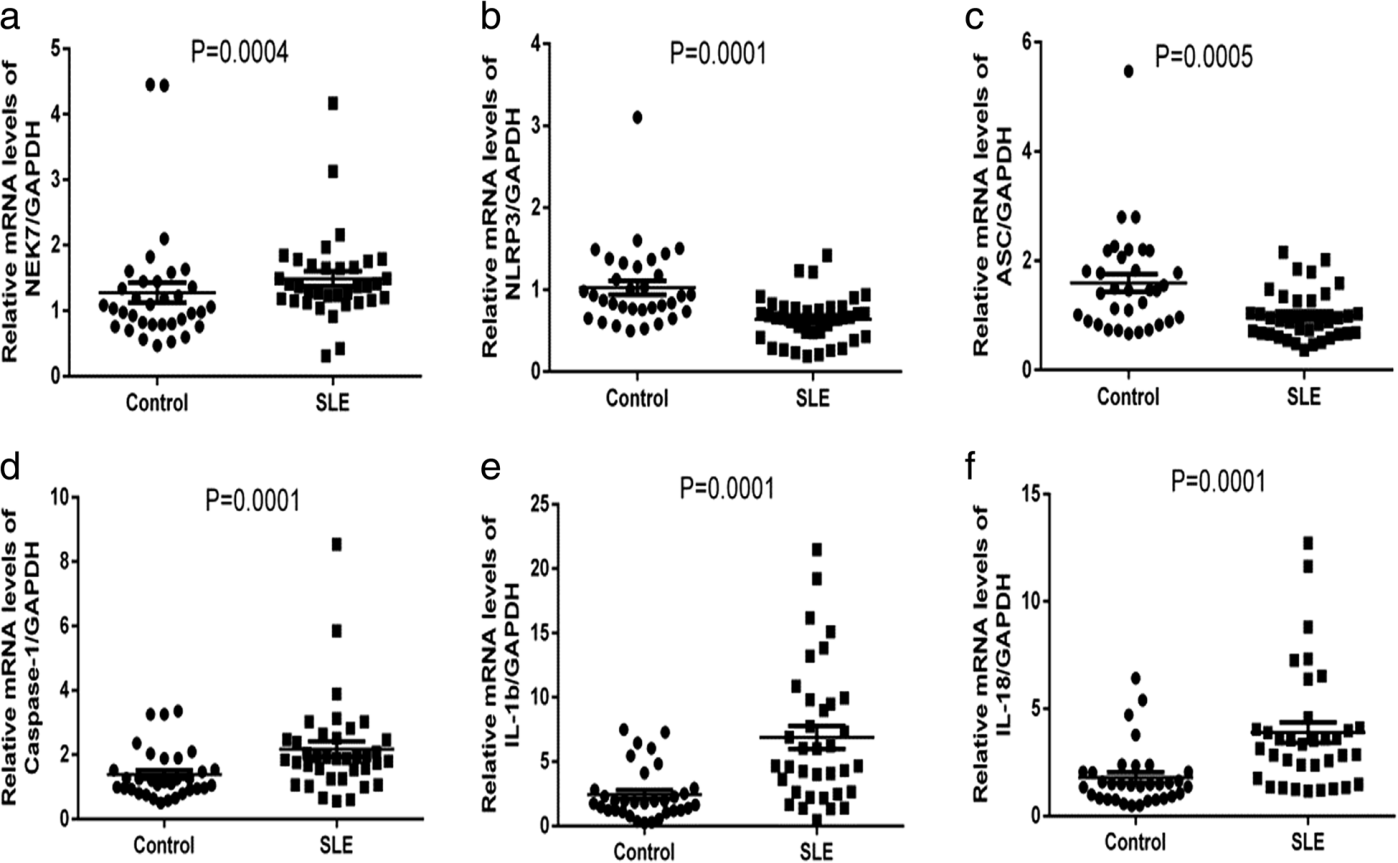
Levels of mRNA encoding *NEK7*, NLRP3 inflammasome components, and downstream cytokines in peripheral blood mononuclear cells (PBMCs) from systemic lupus erythematosus (SLE) patients and healthy controls, as examined by RT-PCR and statistically analyzed by the Mann-Whitney U test. SLE patients showed lower mRNA levels of *NEK7* (**a**), *NLRP3* (**b**), and *ASC* (**c**), but higher levels of *Caspase-1* (**d**), *IL-1b* (**e**), and *IL-18* (**f**)

The mRNA expression of *NEK7*, NLRP3 inflammasome components (*NLRP3*, *ASC*, *Caspase-1*), and downstream cytokines (*IL-1b*, *IL-18*) in PBMCs from SLE patients and healthy controls was detected by real time-PCR. Results showed that compared to those in healthy controls, level*s* of *NEK7*, *NLRP3*, and *ASC* were lower (*p* = 0.0004, Fig. 1a; *p* = 0.0001, Fig. 1b; *p* = 0.0005, Fig. 1c, respectively), whereas *Caspase-1*, *IL-1b*, and *IL-18* were higher in SLE patients (*p* = 0.0001, Fig. 1d; *p* = 0.0001, Fig. 1e; *p* = 0.0001, Fig. 1f, respectively).

### Protein levels of *NEK7*, NLRP3 inflammasome components, and downstream cytokines in PBMCs from SLE patients and healthy controls

Protein levels in PBMCs were further analyzed by western blotting.

**Fig. 2:**
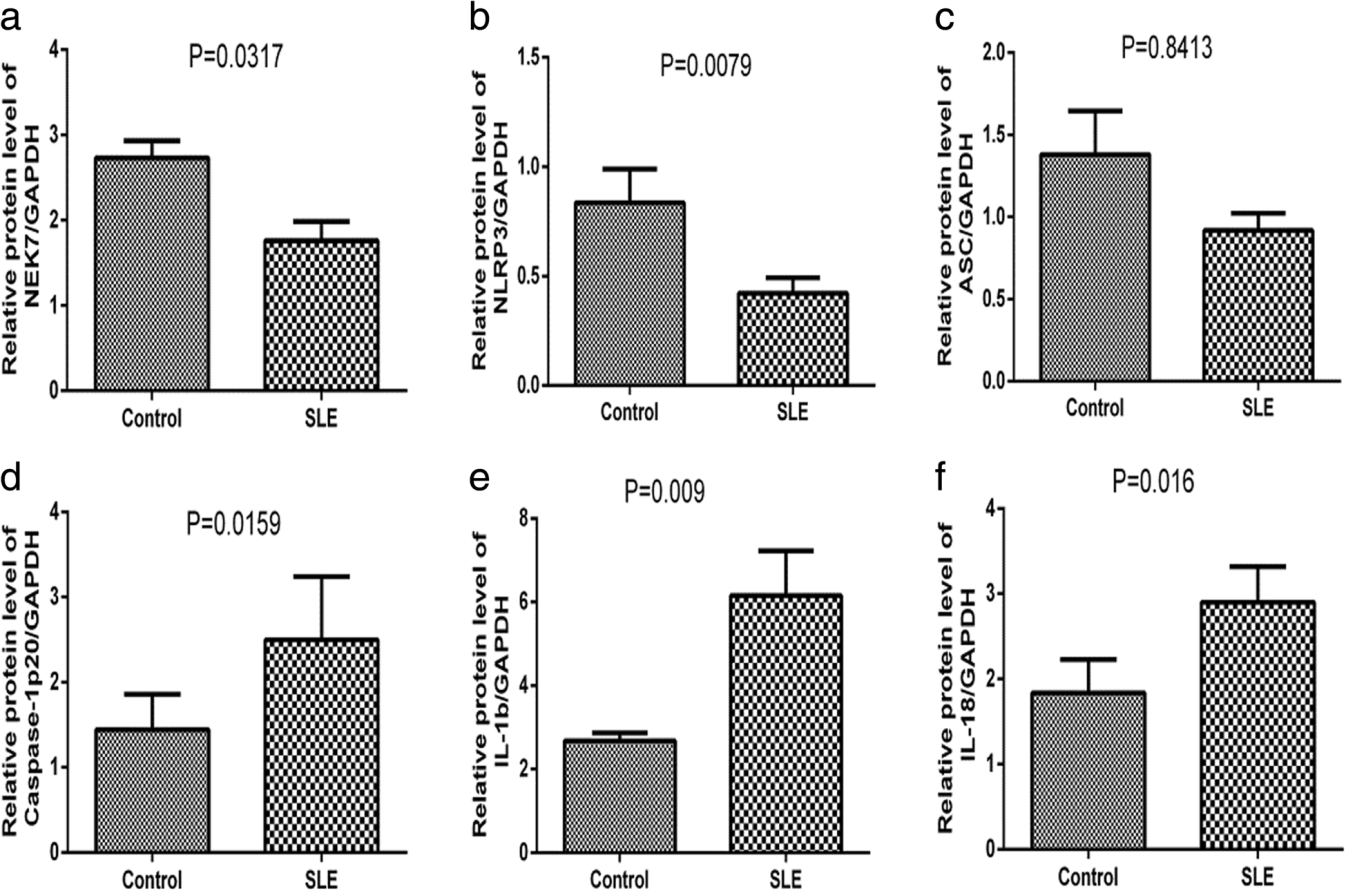
Protein levels of *NEK7*, NLRP3 inflammasome components, and downstream cytokines in peripheral blood mononuclear cells (PBMCs) from systemic lupus erythematosus (SLE) patients and healthy controls, as examined by western blotting and statistically analyzed by the Mann-Whitney U test. Compared to those in healthy controls, SLE patients showed lower protein levels of *NEK7* and *NLRP3*, but higher levels of *Caspase-1, IL-1b* and *IL-18. NEK7* (**a**); *NLRP3* (**b**); *ASC* (**c**); *Caspase-1* (**d**); *IL-1b* (**e**); *IL-18* (**f**)

**Fig. 3:**
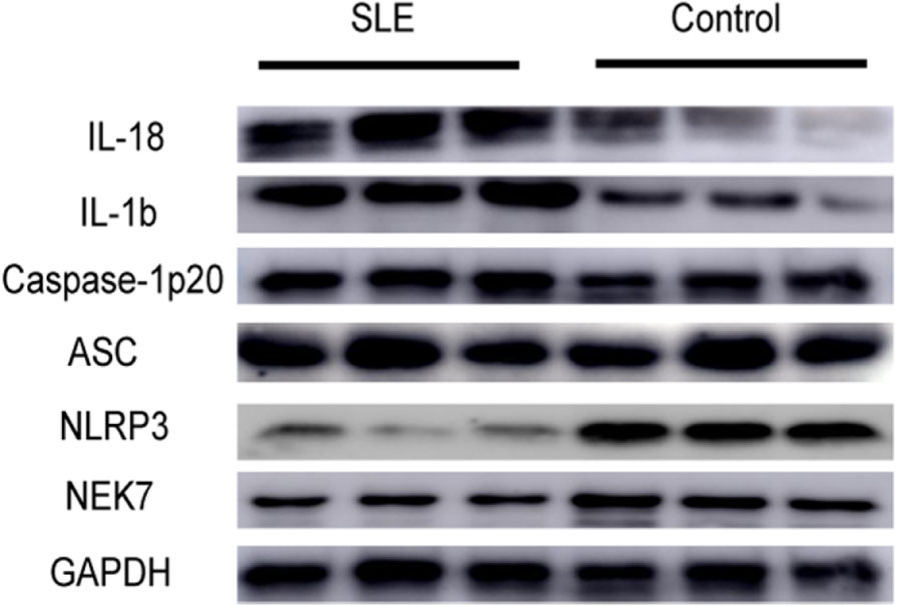
Typical western blotting results of *NEK7*, NLRP3 inflammasome components, and downstream cytokines in SLE patients and healthy controls

Results demonstrated that levels of *NEK7* and *NLRP3* were lower in PBMCs from SLE patients (*p* = 0.0317, Fig. 2a; *p* = 0.0079, Fig. 2b, respectively).However, there were no differences in ASC levels between the two groups (*p* = 0.8413, Fig. 2c). Moreover, *Caspase-1, IL-1b,* and *IL-18* levels were higher in PBMCs from this cohort (*p* = 0.0159, Fig. 2d; *p* = 0.009, Fig. 2e; *p* = 0.016, Fig. 2f, respectively). Typical western blotting results are shown in Fig. 3.

### Serum levels of *IL-1b* and *IL-18* in SLE patients and healthy controls

**Fig. 4:**
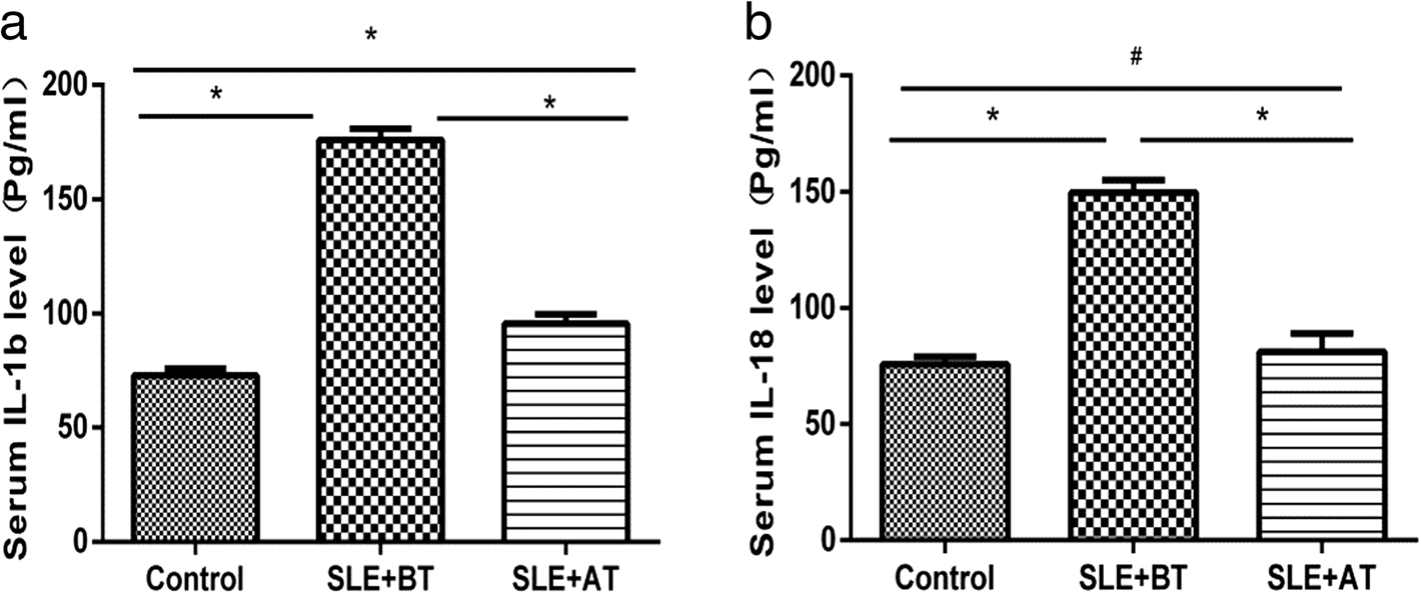
Serum levels of *IL-1b* and *IL-18* in systemic lupus erythematosus (SLE) patients as examined by ELISA. Serum *IL-1b* and *IL-18* levels were higher in the SLE group than in the healthy control group, and the expression of *IL-1b* and *IL-18* was decreased after treatment (continuous methylprednisolone treatment; 40 mg/day for 2 weeks). SLE + BT: SLE patients before treatment; SLE + AT: SLE patients after treatment; *IL-1b* (**a**); *IL-18* (**b**).*p < 0.05, #*p* > 0.05, as determined by the t-test

Results showed that compared to that in healthy controls, *IL-1b* and *IL-18* were highly expressed in SLE patients (*p* = 0.0000, Fig. 4). Moreover, after treatment, serum levels of *IL-1b* and *IL-18* in the SLE group decreased obviously (*p* = 0.0000, Fig. 4); however, in post-treatment SLE patients, *IL-1b* was still elevated compared to that in healthy controls (*p* = 0.0020, Fig. 4a), whereas *IL-18* levels were not significantly different (*p* = 0.4950, Fig. 4b).

### Correlations among *NEK7*, NLRP3 inflammasome components, and downstream cytokine levels in PBMCs from SLE patients

**Fig. 5:**
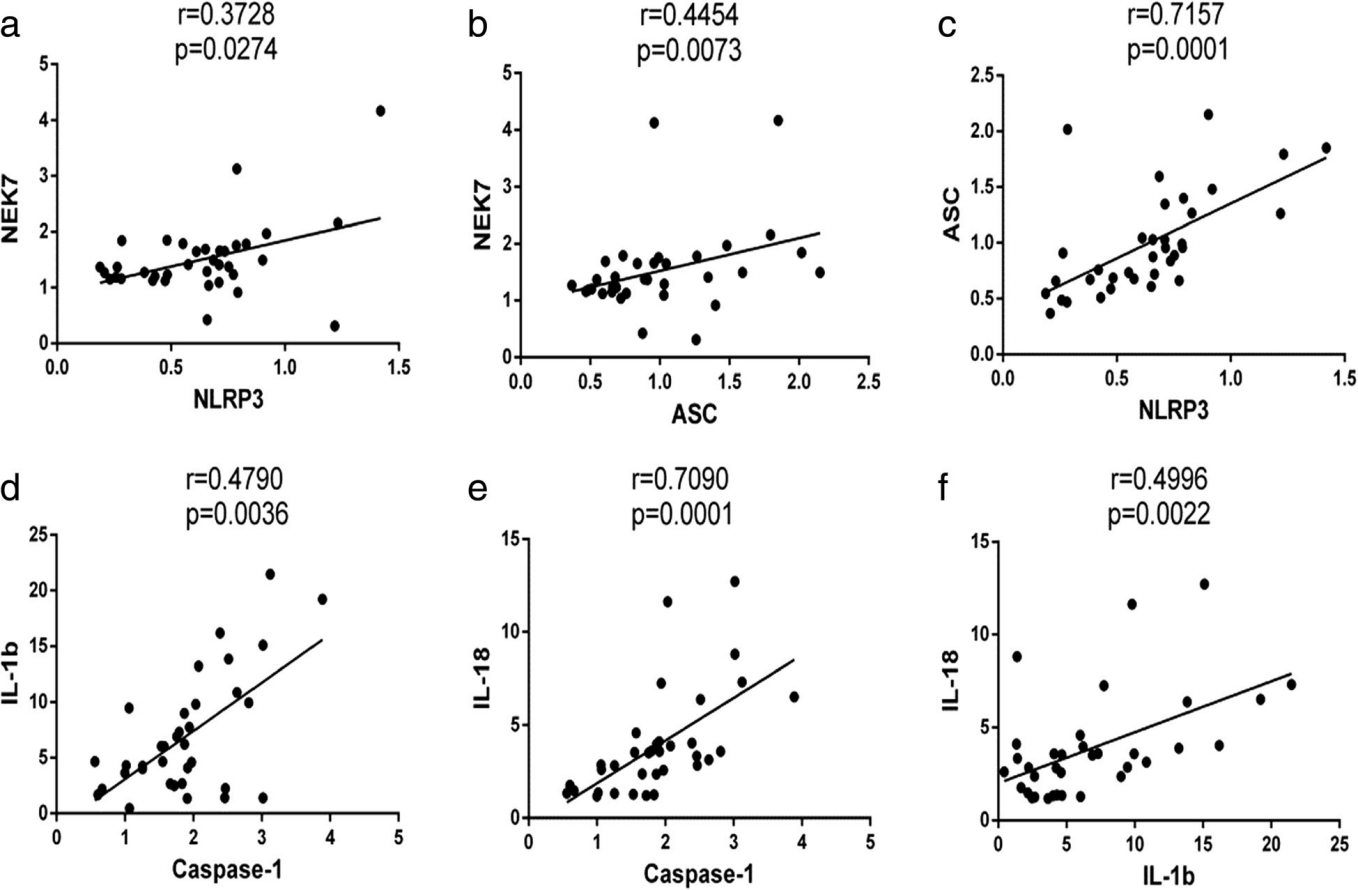
Correlation analysis of mRNA levels of *NEK7, NLRP3* inflammasome components, and downstream cytokines in systemic lupus erythematosus (SLE) patients, only showing statistically significant results. *NEK7* and *NLRP3* (**a**), *NEK7* and *ASC* (**b**), *ASC* and *NLRP3* (**c**), *IL-1b* and *Caspase-1*(**d**), *IL-18* and *Caspase-1*(**e**), *IL-18* and *IL-1b* (**f**)

Correlation analysis comparing mRNA levels of *NEK7*, NLRP3 inflammasome components, and downstream cytokines in PBMCs from SLE patients showed that there was a positive correlation among mRNA levels of *NEK7*, *NLRP3*, and *ASC* (*r* = 0.3728, *p* = 0.0274, Fig. 5a; *r* = 0.4454, *p* = 0.0073, Fig. 5b, respectively), but that *NEK7* mRNA did not correlate with *Caspase-1*, *IL-1b*, and *IL-18* levels (results not shown). There was also a positive correlation between mRNA levels of *NLRP3* and *ASC* (*r* = 0.7157, *p* = 0.0001, Fig. 5c); however, *NLRP3* mRNA levels did not correlate with *Caspase-1*, *IL-1b*, and *IL-18* levels. Correlation analysis also showed that mRNA expression levels of *ASC* did not correlate with *Caspase-1*, *IL-1b*, and *IL-18* levels. mRNA levels of *Caspase-1* positively correlated with those of *IL-1b* and *IL-18* (*r* = 0.4790, *p* = 0.0036, Fig. 5d; *r* = 0.7090, *p* = 0.0001, Fig. 5e, respectively); there was also a positive correlation between the expression levels of *IL-1b* and *IL-18* mRNA (*r* = 0.4996, *p* = 0.0022, Fig. 5f).

### Correlation between mRNA levels of *NEK7*, NLRP3 inflammasome components, and downstream cytokines and SLE disease index

**Fig. 6:**
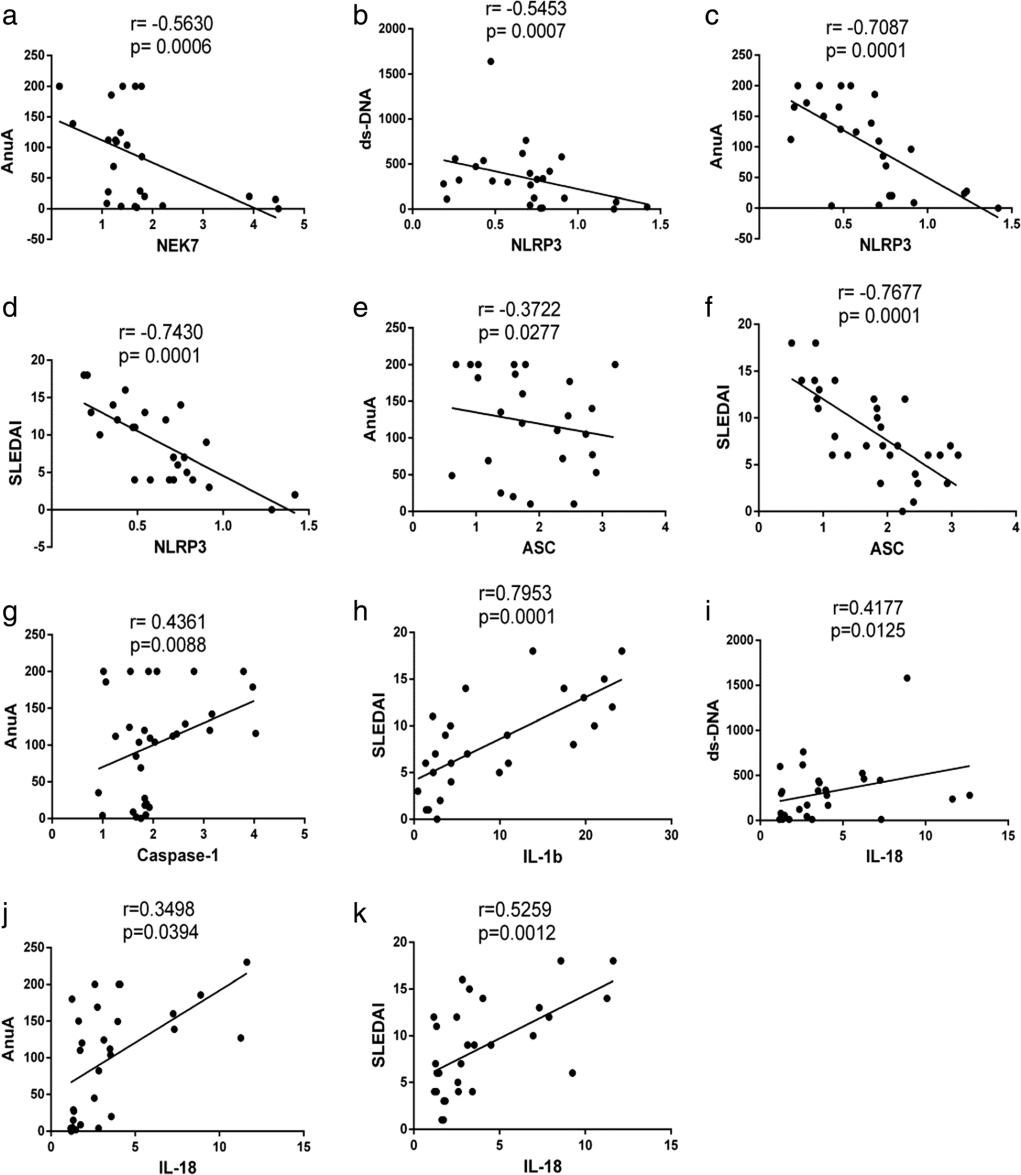
Correlation analysis between the mRNA levels of *NEK7*, NLRP3 inflammasome components (*NLRP3*, *ASC* and *Caspase-1*), its downstream cytokines (*IL-1b* and *IL-18*) and SLE disease activity index (anti-dsDNA, anti-AnuA and SLEDAI). only showing statistically significant results. *NEK7* and AnuA (**a**), *NLRP3* and ds-DNA (**b**), *NLRP3* and AnuA (**c**), *NLRP3* and SLEDAI (**d**), *ASC* and AnuA (**e**), *ASC* and SLEDAI (**f**), *Caspase-1* and AnuA (**g**), *IL-1b* and SLEDAI (**h**), *IL-18* and ds-DNA (**i**), *IL-18* and AnuA (**j**), *IL-18* and SLEDAI (**k**)

Anti-double stranded deoxyribonucleic acid antibodies (anti-dsDNA), anti-nucleosome antibodies (anti-AnuA), and SLEDAI are the common disease activity indices of SLE. Results showed the mRNA levels of *NEK7* were inversely correlated with anti-AnuA (*r* = − 0.5630, *p* = 0.0006, Fig. 6a) but did not correlate with anti-dsDNA or SLEDAI (results not shown). *NLRP3* mRNA levels were inversely correlated with anti-dsDNA, anti-AnuA, and SLEDAI (*r* = − 0.5453, *p* = 0.0007, Fig. 6b; *r* = − 0.7087, *p* = 0.0001, Fig. 6c; *r* = − 0.7430, *p* = 0.0001, Fig. 6d, respectively). *ASC* mRNA levels were inversely correlated with anti-AnuA and SLEDAI (*r* = − 0.3722, *p* = 0.0277, Fig. 6e; *r* = − 0.7667, *p* = 0.0001, Fig. 6f, respectively), but not with anti-dsDNA levels. *Caspase-1* mRNA levels did not correlate with anti-dsDNA or SLEDAI, but positively correlated with anti-AnuA (*r* = 0.4361, *p* = 0.0088, Fig. 6g). *IL-1b* mRNA levels did not correlate with anti-dsDNA or anti-AnuA, but positively correlated with SLEDAI (*r* = 0.7953, *p* = 0.0001, Fig. 6h). *IL-18* mRNA levels were positively correlated with anti-dsDNA, anti-AnuA, and SLEDAI (*r* = 0.4177, *p* = 0.0125, Fig. 6i; *r* = 0.3498, *p* = 0.0394, Fig. 6j; *r* = 0.5259; *p* = 0.0012, Fig. 6k, respectively).

### Correlation between serum levels of *IL-1b* and *IL-18* and SLE characteristics

Serum levels of *IL-1b* in SLE patients were positively correlated with anti-dsDNA, anti-AnuA, ESR, and SLEDAI, and were inversely associated with C3 (*r* = 0.584, 0.504, 0.566, 0.624 and *p* = 0.002, 0.010, 0.003, 0.001, respectively). Serum levels of *IL-18* in SLE patients were positively correlated with anti-dsDNA, ESR, and SLEDAI (*r* = 0.544, 0.512, 0.612 and *p* = 0.005, 0.009, 0.010, respectively; Table 2).

**Table 2:** Correlation analysis between serum levels of IL-1b, IL-18 and SLE laboratory characteristics

Variables	ds-DNA	AnuA	C3	C4	ESR	SLEDAI
IL-1b	0.584^**^	0.504^*^	−0.398^*^	−0.035	0.566^**^	0.624^**^
IL-18	0.544^**^	0.388	−0.376	−0.161	0.512^**^	0.612^*^

Spearman rank correlation analysis: ^*^p < 0.05; ^**^p < 0.01; ^***^p < 0.001

### Changes in mRNA levels of *NEK7*, NLRP3 inflammasome components, and downstream cytokines in SLE patients after treatment

**Fig. 7:**
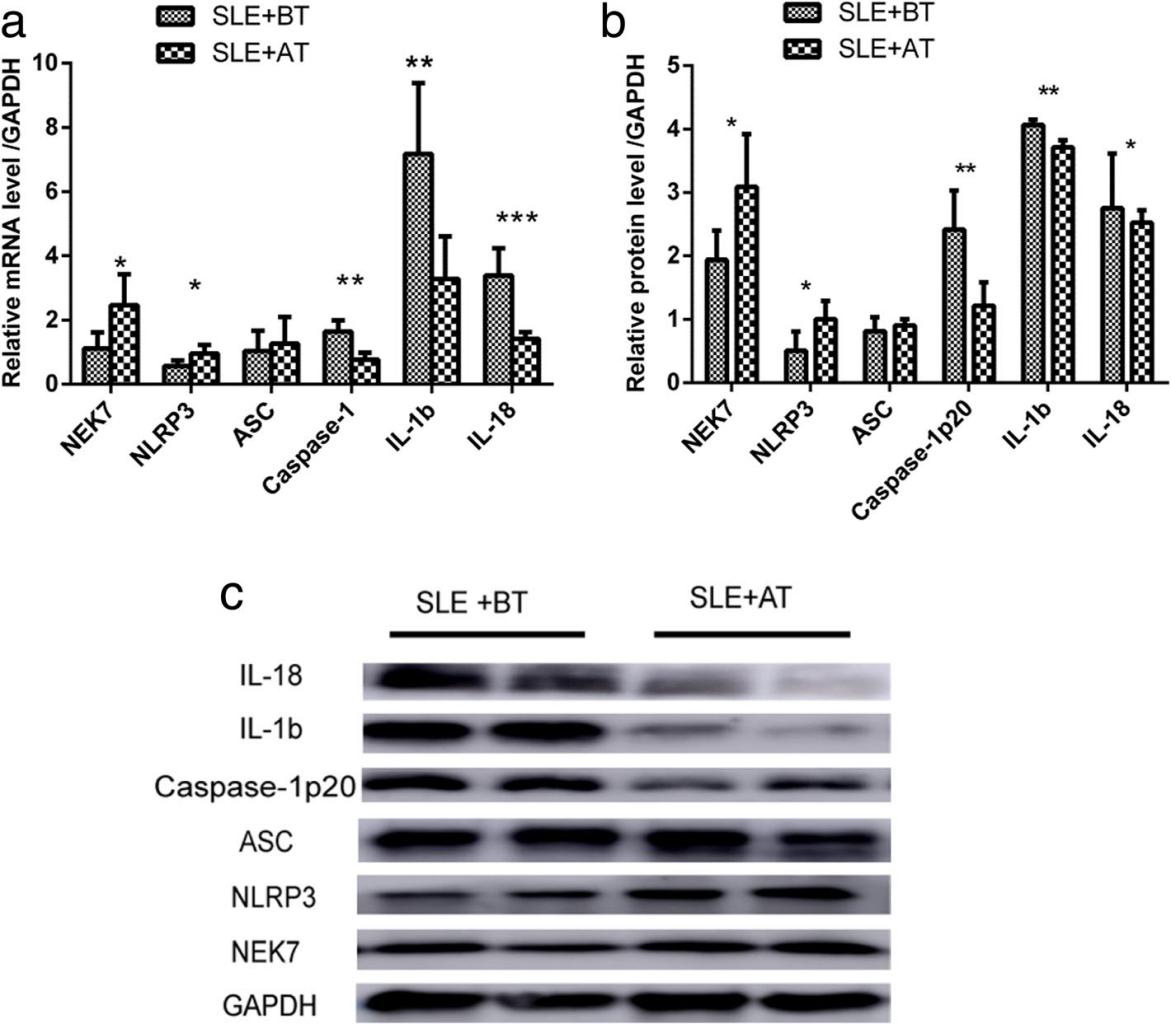
mRNA and protein levels of NEK7, NLRP3 inflammasome components, and downstream cytokines in patients with systemic lupus erythematosus (SLE) after treatment (continuous methylprednisolone treatment; 40 mg/day for two weeks); mRNA levels (**a**) protein levels (**b**) and typical western blotting results (**c**). SLE+BT: SLE patients before treatment; SLE+AT: SLE patients after treatment. *p < 0.05,**p < 0.01, ***p < 0.0001

After drug treatment (methylprednisolone 40 mg/day, 2 weeks), the mRNA levels of *NEK7* and *NLRP3* increased obviously (1.1189 ± 0.5017 vs 2.4685 ± 0.9712, *p* = 0.025 for NEK7; 0.5726 ± 0.1718 vs 0.9682 ± 0.2675, *p* = 0.024 for NLRP3). However, there was no significant difference in *ASC* mRNA levels before and after treatment (1.0350 ± 0.6351 vs 1.2686 ± 0.8319, *p* = 0.634). The mRNA expression of *Caspase-1*, *IL-1b*, and *IL-18* decreased significantly after treatment (1.6419 ± 0.3514 vs 0.7687 ± 0.2223, *p* = 0.002 for Caspase-1; 7.1753 ± 2.2051 vs 3.2899 ± 1.3161, *p* = 0.010 for IL-1b; 3.3933 ± 0.8431 vs 1.4175 ± 0.2151, *p* = 0.001 for IL-18; Fig. 7a).

### Changes in protein levels of *NEK7*, NLRP3 inflammasome components, and downstream cytokines in SLE patients after drug treatment

After drug treatment, the protein levels of *NEK7* and *NLRP3* increased significantly (1.9415 ± 0.4547 vs 3.0900 ± 0.8325, *p* = 0.027 for *NEK7* and 0.5056 ± 0.3042 vs 1.0066 ± 0.2865, *p* = 0.046 for *NLRP3*). However, there was no significant difference in *ASC* protein levels in SLE patients before and after treatment (0.8152 ± 0.2222 vs 0.9048 ± 0.1032, *p* = 0.437). The expression of *Caspase-1*, *IL-1b*, and *IL-18* decreased significantly after treatment (2.4149 ± 0.6172 vs 1.2169 ± 0.3724, *p* = 0.006 for *Caspase-1*; 4.0684 ± 0.0855 vs 3.7134 ± 0.1129, *p* = 0.001 for *IL-1b*; 2.7540 ± 0.8599 vs 2.5211 ± 0.1986, *p* = 0.043 for *IL-18*; Fig. 7b. Typical western blotting results are shown in Fig. 7c.

### Changes in *NEK7*, NLRP3 inflammasome components, and downstream cytokines between the LN group and SLE patients without kidney damage

**Fig. 8:**
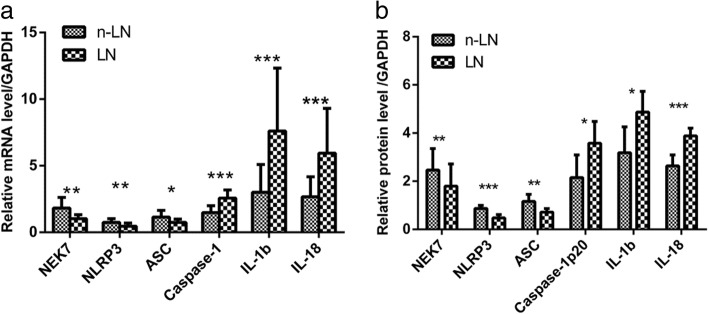
mRNA and protein expression levels of *NEK7*, NLRP3 inflammasome components, and downstream cytokines in patients with lupus nephritis and systemic lupus erythematosus (SLE) patients without renal damage. **a** mRNA levels; **b** protein levels. LN: lupus nephritis; n-LN: SLE patients without lupus nephritis. *p < 0.05, **p < 0.01, ***p < 0.0001

Compared to those in PBMCs from SLE patients without renal damage, patients with LN exhibited lower mRNA and protein levels of *NEK7*, *NLRP3*, and *ASC* (1.8173 ± 0.7953 vs 1.0187 ± 0.3036, *p* = 0.002 for *NEK7* mRNA; 0.7432 ± 0.2791 vs 0.4612 ± 0.2317, *p* = 0.004 for *NLRP3* mRNA; 1.1426 ± 0.4972 vs 0.7455 ± 0.2536, *p* = 0.012 for *ASC* mRNA; 2.4661 ± 0.8916 vs 1.7945 ± 0.9244, *p* = 0.010 for *NEK7* protein; 0.8669 ± 0.1290 vs 0.4779 ± 0.1400, *p* = 0.001 for *NLRP3* protein; 1.1628 ± 0.2963 vs 0.7191 ± 0.1434, *p* = 0.008 for *ASC* protein), and higher mRNA and protein levels of *Caspase-1*, *IL-1b*, and *IL-18* (1.4763 ± 0.5189 vs 2.5654 ± 0.6186, *p* = 0.000, for *Caspase-1* mRNA; 3.0022 ± 2.0904 vs 7.6056 ± 4.7246, *p* = 0.000 for *IL-1b* mRNA; 2.6668 ± 1.4962 vs 5.9491 ± 3.3549, *p* = 0.000 for *IL-18* mRNA; 2.1471 ± 0.9455 vs 3.5734 ± 0.9081, *p* = 0.024 for *Caspase-1* protein; 3.1865 ± 1.0688 vs 4.8724 ± 0.8605, *p* = 0.013 for *IL-1b* protein; 2.6391 ± 0.4480 vs 3.8800 ± 0.3223, *p* = 0.000 for *IL-18* protein; Fig. 8).

## Discussion

Previous studies have found that there are four main pathways of NLRP3 inflammasome activation. First, in the potassium efflux model, metabolites and endotoxin disrupt the integrity of the cell membrane or the binding of ATP to the P2X7 receptor on the cell membrane, leading to the opening of specific potassium channels, intracellular potassium efflux, and a disruption of mitochondrial structure and function, eventually activating the NLRP3 inflammasome [10, 11]. The second is the direct activation of reactive oxygen species (ROS); here, mitochondrial-derived ROS directly activates the NLRP3 inflammasome and with specific ROS inhibitors, the generation of *IL-1b* was found to be markedly reduced [12]. Third, substances such as crystals or particles entering the cell induce the destruction of corpuscles and the release of cathepsin, thereby activating the NLRP3 inflammasome [13]. Fourth, metabolites such as fatty acids, peptides, and toxins can also activate the NLRP3 inflammasome by coating microporous structures [14].In addition to the combined effects of one or more of these pathways, the activation of the NLRP3 inflammasome also requires the synergistic action of nuclear transcription factor kappa B (NF-κB) signaling pathways and the modification of NLRP3 by ubiquitination. In conclusion, activation of the NLRP3 inflammasome is a complex process that is regulated by many factors and pathways [15]. In 2016, Gabriel et al. [9] found that *NEK7* plays an important role in the efflux of intracellular potassium ions, and that it is a key protein that activates the NLRP3 inflammasome. The NOD domains and leucine rich repeat (LRR) of NLRP3 components might be the key sites for these interactions. In addition, the lack of *NEK7* also specifically blocks activation of the NLRP3 inflammasome. Studies have also found that murine macrophages deficient in NEK7 exhibit a diminished response to lipopolysaccharide (LPS) stimulation. Moreover, compared to wild-type mice, *NEK7*-deficient mice with multiple sclerosis present with fewer *IL-1b*-related inflammatory diseases [16]. Schmid-Burgk et al. [17] used the clustered regularly interspaced short palindromic repeats (CRISPR/Cas9) system to detect murine macrophage strains, and found that *NEK7*-knockout cells show reduced sensitivity to apoptosis induced by nigericin, mediated by low *Caspase-1* and *IL-1b* expression. Current studies have also shown that *NEK7* plays a crucial role in the activation of the NLRP3 inflammasome, but most studies have been carried out in mice and in vitro, and there have been no reports of associated disease studies in humans. The purpose of this study was to investigate the expression and clinical significance of NEK7–NLRP3 signaling in Chinese Han SLE patients.

*NEK7*, as a member of the NIMA-related protein kinase family, concentrates at the poles of the spindle, functions to regulate microtubules, is closely related to the formation of the mitotic spindle and separation of the cytoplasm, and plays an important role in the regulation of cell cycle [18]. *NEK7* and NEK6 are highly homologous; both are not only structurally similar but also serve as a common substrate for NEK9 and are functionally synergistic. NEK6, 7, and 9 comprise an important group of proteins in which all three exert synergistic effects. In its absence or with abnormal expression of *NEK7*, cell mitosis is blocked, which results in apoptosis. Presently, research on *NEK7* has been mainly concentrated in the field of malignant tumorigenesis, and this protein is closely related to the occurrence of breast and cervical cancer [19]. The combination of *NLRP3* and *ASC* can lead to mitochondrial structural damage and acetylation of microtubules, and the main function of *NEK7* is to maintain the dynamic stability of microtubule structure. It has also been indirectly shown that *NEK7* is closely related to activation of the NLRP3 inflammasome. The conserved and extensive expression of *NEK7* and its importance in mitosis suggest that it is not a specific NLRP3 inflammasome-activating protein [17]. Studies have found that during interphase, the NLRP3 inflammasome can be activated by substances such as LPS, ATP, and nigericin, whereas in mitosis, activation of the NLRP3 inflammasome was markedly inhibited under the same stimulation. This shows that the *NEK7*-mediated regulation of cell mitosis and NLRP3 inflammasome activation are mutually exclusive events, and that neither can occur simultaneously [16]. Similarly, compared to that in mitotic cells, the NEK7–NLRP3 complex is highly expressed in interphase cells [20]. Thus, the expression of this complex in cells is related to growth cycle.

In this study, the mRNA and protein expression of *NEK7*, *NLRP3*, and *ASC* in PBMCs from SLE patients was significantly lower than that in cells from healthy controls, and the difference was statistically significant. Moreover, the mRNA expression of *NEK7*, *NLRP3*, and *ASC* was inversely correlated with the disease activity index of SLE. Interclass correlation analysis showed that the mRNA expression of *NEK7*, *NLRP3*, and *ASC* were positively correlated. These results suggest that *NEK7* activates the NLRP3 inflammasome in PBMCs from SLE patients, and that the NEK7–NLRP3 complex is closely related to the immune and inflammatory responses observed in SLE. For patients with SLE, after continuous methylprednisolone treatment (40 mg/day for 2 weeks), SLEDAI scores were reduced, clinical symptoms were relieved, and the mRNA and protein expression levels of *NEK7*, *NLRP3*, and *ASC* in PBMCs from SLE patients were up-regulated, suggesting that the NEK7–NLRP3 complex might act as a protective factor during the pathogenesis of SLE.

The low expression of the NLRP3 inflammasome in PBMCs from SLE patients might be related to the direct inhibitory effect of the adaptive immune system on the activation of T cells and the inhibition NLRP3 by interferon via the STAT1 signaling pathway. It might also be associated with the low expression of *NEK7* in patients with SLE. In addition, the mRNA and protein expression levels of *NEK7*, *NLRP3*, and *ASC* in LN patients were significantly lower than those in SLE patients without kidney damage, suggests that this is closely related to the occurrence of LN and could be a protective factor involved in the pathogenesis of LN.

The mRNA and protein expression of *Caspase-1* in PBMCs from SLE patients was significantly higher than that in healthy controls, and was positively correlated with SLE disease activities. After treatment, the expression of *Caspase-1* decreased significantly. In addition, the mRNA and protein expression of *Caspase-1* in the LN group was significantly higher than that in SLE patients without kidney damage. This reveals that high *Caspase-1* expression is associated with the pathogenesis of SLE and LN and is positively related to disease activity. These results were in conflict with the observed low expression of *NEK7*, *NLRP3*, and *ASC*, and suggests that the expression of *Caspase-1* in PBMCs from SLE patients is not only affected by NLRP3 inflammasome signaling, but also by other signaling pathways.

Consistent with our experimental results, Zhang et al. [21] reported that compared to those in healthy controls, the expression levels of *Caspase-1* and *IL-1b* in patients with SLE were significantly increased and positively correlated with disease activity. Moreover, they further confirmed that in patients with SLE, ds-DNA interacts with Toll like receptor 4 (TLR4) and induces the mitochondrial production of ROS in monocytes and macrophages, eventually leading to high *Caspase-1* expression. As is known, *Caspase-1* is widely involved in cell growth, differentiation, injury, repair, and apoptosis, and it is differentially expressed in diverse contexts [22]. Furthermore, a large number of apoptotic bodies and abnormal apoptosis have been observed in PBMCs from SLE patients, which might be related to the high expression of *Caspase-1* [23]. Experiments in mouse models of lupus have shown that Caspase-1P20 is activated, and that inhibition of this activation can significantly reduce infection and improve disease severity [24]. In a mouse model of LN, the severity of clinical signs was markedly improved by specific *Caspase-1* inhibitors [25]. However, in our previous study, *Caspase-1* was found to be poorly expressed in SLE patients, and the results were inconsistent. Possible reasons for this are as follows. First, in the early stage of disease, *Caspase-1* is highly expressed and induces the maturation and release of downstream factors; as a risk factor, it is involved in immune and inflammatory reactions in the organism. When the disease develops, *IL-1b* and *IL-18* cluster together to activate programmed cell apoptosis, which is dependent on the *Caspase-1* pathway via feedback. Comparing these studies, the two groups had different disease periods, which might be the main reason for these inconsistent results. Second, the two groups had different degrees of disease activity. In our previous study, the mean SLEDAI score of patients with SLE was 15 (2–26), and in this study, it was 8.4 (0–18); thus, there were significant differences in disease activity between the two groups. Differences might also be related to individual differences, experimental errors, and other factors.

In our study, the mRNA and protein expression of *IL-1b* and *IL-18* in PBMCs from SLE patients was significantly higher than that in healthy controls, and this was positively correlated with disease activity. Further, after treatment, the expression of these markers decreased obviously. Similarly, serum levels of *IL-1b* and *IL-18* in patients with SLE were also high and positively correlated with the evaluation index of SLE disease activities; these levels were also significantly decreased after drug treatment. The mRNA and protein expression of *IL-1b* and *IL-18* in the LN group was significantly higher than that in the SLE group without kidney damage, suggesting that high expression of these markers is closely related to the incidence of SLE and LN, because high activation of *Caspase-1* eventually leads to high expression of *IL-1b* and *IL-18*. Wang et al. [26] found that in patients with SLE, *IL-1b* was highly expressed and involved in multiple organ damage during SLE, and was especially associated with the occurrence of neuropsychiatric lupus. The fact that it is difficult to induce lupus in *IL-1b*-deficient mice indirectly suggests that *IL-1b* has an irreplaceable role in the pathogenesis of SLE [27]. The possible role of *IL-1b* in the pathogenesis of SLE might also be related to the activation of B cells and the production of immunoglobulin IgG and autoantibodies such as anti-ds-DNA [28]. In addition, anakinra, an IL-1 receptor antagonist, can effectively reduce clinical symptoms and disease severity in patients with SLE [29, 30]. One study found that compared to that in healthy controls, the expression of *IL-18* in the serum of SLE patients increased significantly. In patients with LN, this increase was more pronounced, and the serum levels of *IL-18* were positively correlated with SLEDAI score and urinary proteins [31, 32]. In addition, *IL-18* gene polymorphisms were found to be significantly associated with the occurrence of arthritis symptoms in SLE patients [33]. The role of *IL-18* in the development of SLE might be related to the induction of other proinflammatory cytokines such as TNF-α, IFN-γ, and IL-1. This suggests that the activation and release of *IL-1b* and * IL-18*, mediated by excessive activation of *Caspase-1* in PBMCs from SLE patients, contribute to the pathogenesis of SLE and LN.

## Conclusions

This study demonstrated that *NEK7* positively activates *NLRP3* in PBMCs from patients with SLE, and that low expression of *NLRP3* might be related to the low expression of *NEK7*. The expression of the NEK7–NLRP3 complex could be involved in the pathogenesis of SLE and LN as a protective factor; moreover, this complex might represent a new target for the treatment of SLE.

## Methods

### Materials

Thirty-eight SLE patients (SLE group) were enrolled from the Department of Rheumatology and Immunology in Shandong Provincial Hospital affiliated to Shandong University, from March to November 2016. The inclusion criteria were as follows: in accordance with the American College of Rheumatology classification criteria (ACR 2009) for SLE and did not receive drug treatments for approximately 6 months [4]; the exclusion criteria were as follows: patients with liver or kidney disease, infection, cancer, metabolic diseases, and other autoimmune diseases. A follow-up study of 25 cases of SLE patients after treatment (methylprednisolone 40 mg/day for 2 weeks) was also conducted. The control group was composed of 33 healthy volunteers from the physical examination center of the hospital during the same period (healthy control group), and there were no abnormalities based on hematology and physical examination in the control group. The two groups were well matched for age, sex, and race (*p* > 0.05).

### Methods

#### Extraction of PBMCs

Twenty milliliters of fasting blood was taken from the subjects with heparin anticoagulation. The PBMC layer was obtained by Ficoll gradient centrifugation (Lymphoprep, Nycomed Pharma AS) according to manufacturer’s instructions. PBMCs were eventually obtained by repeated washing with phosphate buffered saline (PBS).

#### RNA extraction and quantitative real-time PCR

Total RNA from PBMCs was extracted using Trizol (Invitrogen) according to the manufacturer’s instructions. The Nanodrop micro photometer was used to determine the concentration and purity of total RNA, and the A260/A280 ratio was between 1.8 and 2.0. Reverse transcription was performed using the PrimeScriptTMRT reagent Kit with gDNA Eraser (Takara) with total RNA as a template. Using GAPDH as a reference and complementary DNA (cDNA) as the template, quantitative Real-Time PCR was performed using SYBR ®Premix Ex TaqTM kit (Takara). Primer sequences of the target and reference genes are as follows: *NEK7* forward 5′-CCA CTG GGA TGG TAA AAC TTG-3′, reverse 5′-AAG GAC TTT GTA ATG CAG CCA T-3′; *NLRP3* forward 5′-CGT GAG TCC CAT TAA GAT GGA GT-3′, reverse 5′-CCC GAC AGT GGA TAT AGA ACA GA -3′; *ASC* forward 5′- TGG ATG CTC TGT ACG GGA AG-3′, reverse 5′-CCA GGC TGG TGT GAA ACT GAA-3′; *Caspase-1* forward 5′-TTT CCG CAA GGT TCG ATT TTC A -3′, reverse 5′-GGC ATC TGC GCT CTA CCA TC -3′; *IL-1b* forward 5′-TTC GAC ACA TGG GAT AAC GAG G-3′, reverse 5′-TTT TTG CTG TGA GTC CCG GAG-3′; *IL-18* forward 5′-TCT TCA TTG ACC AAG GAA ATC GG-3′, reverse 5′-TCC GGG GTG CAT TAT CTC TAC-3′; GAPDH forward 5′-GCA CCG TCA AGG CTG AGA AC-3′, reverse 5′-TGG TGA AGA CGC CAG TGG A-3′ (Sangon Biotech). Each sample was examined three times, according to the dissolution curve and the Ct value, the 2^-∆∆CT^ method was used to calculate the relative mRNA expression.

#### Protein extraction and western blotting

Caspase-1 exists in a form without zymogen activity, and when cells are stimulated by a variety of extracellular pathogens or intracellular danger signals, they are assembled to form an inflammasome, which recruits pro-caspase-1 to achieve local high concentrations. At this time, the zymogen undergoes autologous hydrolysis, producing two subunits, p20 and p10, and forming a p20/p10 isomer, followed by another two polymers, which eventually becomes active caspase-1. Therefore, we tested the expression of p20. A mixture of RIPA buffer and PMSF was added, in accordance with the manufacturer’s instructions, to extract protein (Beyotime). The BCA Protein Assay Kit (Beyotime) was used to determine protein concentrations. Proteins were then denatured; the amount of protein per sample is 50 μg, then electrophoretic use of 8% SDS-PAGE gel, and transferred to PVDF membranes, which were subsequently incubated with antibodies. Specific antibodies were as follows: anti-*NEK7* antibody (#3057, Cell Signaling Technology, 1:1000 dilution); anti-*NLRP3* and *IL-1b* antibody (#NM-001127461 and #16806–1, Proteintech, 1:1000 dilution, respectively); anti-*ASC* antibody (#sc-514,414, Santa Cruz, 1:100 dilution); anti-*Caspase-1p20 *and *IL-18* antibody (#ab207802 and #ab207324, Abcam, 1:1000 and 1:200 dilution, respectively); GAPDHwas used as the reference (#sc-25,778, Santa Cruz, 1:1000 dilution). After samples were exposed and developed using the Automatic gel imaging analysis system (Gene Genius), Image J software was used for data analysis, and band intensity was normalized to that of GAPDH.

#### Serum extraction and enzyme-linked immunosorbent assays (ELISAs)

Serum was obtained after centrifugation (1000×*g*, 20 min) and stored at − 80 °C until analyses. The ELISA double resistance method was used to measure the concentrations of *IL-1b* and *IL-18* in samples following the manufacturer’s instructions (Boster Biological Engineering and Cloud-Clone Corp).

#### Collection of clinical and laboratory indices

The clinical characteristics of subjects were collected, such as age, sex, course of disease, and clinical and laboratory indices. Laboratory indices such as anti-dsDNA antibody, anti-nucleosome antibody (AnuA), erythrocyte sedimentation rate (ESR), Complement 3 (C3), Complement 4 (C4), immunoglobulins (Igs), and proteinuria were analyzed for 24 h by the Department of Clinical Laboratory of Shandong Provincial Hospital. The systemic lupus erythematosus disease activity index (SLEDAI) was obtained by professional clinicians.

### Statistical analysis

Statistical software SPSS (version 19.0) and GraphPad Prism (version 5.0) were used for analysis. Chi-squared tests and t-tests were performed to analyze differences in sex and age distribution. Target mRNA and protein levels were compared using the Mann-Whitney U test. The Spearman test was used to evaluate the correlation between mRNA levels and disease activity indices such as ds-DNA, AnuA, ESR, C3, C4, and SLEDAI. *p* value less than 0.05 was considered statistically significant. All tests were two-tailed.
